# Vaccination against human papillomavirus

**DOI:** 10.1590/S1679-45082013000400027

**Published:** 2013

**Authors:** Claudia Figueiredo Mello

**Affiliations:** 1 Instituto de Infectologia Emílio Ribas, São Paulo, SP, Brazil

**Keywords:** Vaccination, Immunization schedule, Papillomavirus vaccines

## Abstract

Human papillomavirus infection is common and causes different manifestations. This infection is a public health concern because it has been associated with genital tract malignant diseases among men and women. Currently two vaccines are available to prevent the human papillomavirus infection and its associated diseases.

## RESUMO

A infecção pelo papilomavírus humano é comum e produz manifestações diversas. Essa infecção adquire importância para saúde pública, pois tem sido associada a malignidades do trato genital em homens e mulheres. Atualmente, temos disponíveis duas vacinas para prevenção da infecção pelo papilomavírus humano e, consequentemente, de doenças a ele associadas.

## INTRODUCTION

The human papilomavirus (HPV) infection is common and causes several manifestations such as common warts, epidermodysplasia verruciformis, anogenital warts, cervical and vulvar cancer, cancer of the penis, vagina and anus, recurrent respiratory papillomatosis, among others^(1)^.

In most of cases infection with HPV is asymptomatic and autolimited, however, it is a public health concern because of its association with genital tract malignant disease among men and women^(1)^.

HPV isolations are classified in “types” and they receive a number to reflect the temporal order of its characterization. More than 100 types of HPV were identified to date, and of them at least 40 can infect the genital area^(1)^.

In general, one or more particular HPV types are associated with a clinical manifestation. For example, anogenital warts (also called condylomata accuminata) are caused by types 6 and 11, which account for 90% of cases^(1)^.

Cervical cancer is more related with types 16 and 18, which correspond to 70% of cases, however, this condition could be caused by others HPV types (31, 33, 45 and 56)^(1)^.

HPV genital infection is mainly transmitted by genital-to-genital contact often during sexual intercourse. However, other forms of transmissions are reported, *e.g.*, children may acquire genital warts from hand contact with nongenital lesions^(1)^.

The majority of sexually active population will be in contact with HPV at some point in their lives. Condoms' use might reduce the risk for HPV and HPV-associated diseases, but condom protection against this virus is not infallible^(2)^.

## VACCINE TYPES

Another form for HPV prevention is vaccination. Nowadays, there are two types of vaccines against HPV, the quadrivalent HPV (HPV 4) and the vaccine to prevent oncogenic HPV (HPV2). Both vaccines are based on virus-like particles (VLPs) using the recombinant DNA technique, which creates one of the proteins that compose the capsid of HPV, the L1 protein^(2)^.

VLPs can induce generation of high titers neutralizing antibodies that are enough to protect those who are vaccinated. This protection is independent of cell immune response^(2)^.

The HPV4 vaccine contains VLPs that are similar to those found in HPV types 6, 11, 16 and 18. To produce this vaccine, the L1 gene of these genotypes is expressed in *Saccharomyces cerevisiae* (yeast) and is used with an aluminum adjuvant^(2)^.

Clinical studies have shown that HPV4 offer protection against persistent HPV infection, cervical lesions precursor of cancer, vaginal and vulvar lesions precursor of cancer and genital warts caused by HPV types 11, 16 or 18 in women aged 16 to 26 years old and who were not previously infected by these HPV types^(3)^.

A randomized multicenter, prospective, placebo-controlled, double-blind study with healthy boys and men 16 to 26 years of age reported that vaccine was effective to prevent infection of HPV types 6, 11, 16 e 18, besides preventing external genital warts associated with these HPV types in individuals who received HPV4 vaccine^(4)^.

The HPV4 vaccine (Gardasil^TM^, Merck and Co., Inc., Whitehouse Station, New Jersey) is indicated for both sexes aged between 9 to 26 years, and it should be administrated in intervals of 0, 2 and 6 months^(5)^.

HPV2 vaccine contains VLPS similar to those found in HPVs types 16 and 18, produced using the recombinant DNA technique in insect cells. In such cases, the AS04 adjuvant is used^(6)^.

The efficacy of HPV2 vaccine was assessed in two randomized, controlled, double-blind studies composed by women 15 to 25 years of age. These studies concluded that HPV2 vaccine was efficient to prevent cervical cancer precursor lesions caused by HPV types 16 and 18 in women not previously infected with these virus^(7)^.

The HPV2 vaccine (Cervarix^®^, GlaxoSmithKline Biologicals, Rixensart, Belgium) is indicated for girls and women aged 10 to 25 years old, and it should be administrated in intervals of 0, 1 and 6 months^(5)^.

Ideally, HPV vaccine should be administered between 11 to 12 years of age. Vaccination in women over 25 or 26 years is considered safe and efficient by regulatory organs of some countries, although no randomized, controlled studies exist on vaccine administration in women in this age group. Considering this fact, based on medical criterion, women over 25 or 26 years old can be vaccinated^(8)^.

HPV vaccines are contraindicated for persons with a history of allergic reaction to the vaccine or to any of its components. Pregnant women should be vaccinated after pregnancy. The HPV4 is also contraindicated for persons with history of immediate hypersensitivity to *Saccharomyces cerevisiae* (yeast)^(6)^.

Previous infection with HPV does not constitute a contraindication for vaccination, and there are no evidences on protection against diseases caused by HPV types that had already been infected the individual at the time of vaccination, but the vaccine could protect against diseases caused by other HPV types covered by the vaccine^(6)^.

Because this is an inactive, non-infectious vaccine, there is no contraindication for immunosuppressed individuals, although immunogenicity in this group of patients is not guaranteed^(6)^.

Both HPV vaccines have good tolerability profiles and present few adverse effects, the most commonly reported are injection-site pain and swelling^(6)^.

Syncope (faintness) can occur after vaccination and has been observed particularly among adolescents and young adults. To avoid serious injury related to a syncopal episode, vaccine providers should consider observing patients for 15 minutes after vaccination^(6)^.

HPV vaccines can be administered to individuals with mild acute illnesses. Vaccination of persons with moderate or severe acute illnesses should be deferred until after improvement of clinical picture^(6)^.

If the vaccine series is interrupted it could be restarted anytime since the minimal interval between missing doses is followed. Coadministration of different vaccines either simultaneously or at any time before or after HPV vaccine is permitted if different injection-sites are used^(6)^.

The vaccine against HPV is an important tool to prevent infection with HPV and its associated diseases. However, vaccination does not exclude the need of performing the screening for cervical cancer prevention (Papanicolaou's test), because cervical cancer could be related to other HPV types not covered by the vaccine^(1)^.
